# Regional Inequalities in Diagnosis and Therapies in Greece regarding Autism Spectrum Disorders

**DOI:** 10.1192/j.eurpsy.2024.267

**Published:** 2024-08-27

**Authors:** R. Kouznetsov, P. Angelopoulos, S. Moulinos, A. Andrianopoulou, I. Dimakos, P. Gourzis, E. Jelastopulu

**Affiliations:** ^1^Department of Public Health, School of Medicine, University of Patras, Patras; ^2^National and Kapodistrian University of Athens, Athens; ^3^Department of Digital Media and Communication, Ionian University, Corfu; ^4^School of Medicine; ^5^Educational Sciences and Social Work; ^6^Department of Psychiatry, School of Medicine, University of Patras, Patras, Greece

## Abstract

**Introduction:**

Autism spectrum disorders (ASD) represent a major public health concern on a global scale. The increasing prevalence of ASD worldwide, coupled with the arising demand for treatments, underscores its important role in the public mental health discourse. Ensuring the equitable integration of children with ASD and their families into all aspects of society becomes an imperative task, in order to eradicate the stigma associated with the broad spectrum of autism, encompassing both visible and concealed dimensions.

**Objectives:**

The primary objective of this study was to determine the crude prevalence of ASD in Greece nationwide, while also examining regional disparities in both prevalence and therapies. The study spanned a three-year period from February 2019 to February 2022 and relied on retrospective data sourced from the Greek National Organization for Healthcare Services Provision (EOPYY).

**Methods:**

EOPYY provided de-identified data, including information such as sex, age, diagnosis, and treatment for each child, facilitated by hashed social security numbers. Statistical analysis of the dataset was performed using the open-source statistical program R.

**Results:**

A total of 18,245 children aged 2 -17 years were diagnosed with ASD in Greece, representing a nationwide crude prevalence rate of 1.16%. Regional disparities were evident, with prevalence rates ranging from 0.49% in the North Aegean to 1.57% in Crete. Over the three-year study period, a total of 15,328,327 non-medical therapies were prescribed, corresponding to an annual average of 264 therapies per child. Statistically significant differences between the thirteen regions in Greece were observed, ranging from 230 to 323 annual therapies per child.

**Conclusions:**

Our findings align Greece’s ASD prevalence with the global estimate of 1 in 100 children, as per the World Health Organization. Disparities between rural and urban areas in Greece may be attributed to differences in diagnostic procedures and the availability and accessibility of specialized services for autistic individuals. Thus, the establishment of a national surveillance system for ASD is recommended to enhance our understanding of the autism spectrum, monitor changes in prevalence, and identify potential contributing factors to autism conditions. Furthermore, these evidence-based results offer invaluable insights for crafting policies concerning healthcare, education, and employment for individuals with ASD in order to ensure the development of people with autism, their wellbeing, and a good quality of life.

**Disclosure of Interest:**

None Declared

